# Fabrication of Mechanically Enhanced, Suturable, Fibrous Hydrogel Membranes

**DOI:** 10.3390/membranes13010116

**Published:** 2023-01-16

**Authors:** Constantinos Voniatis, Olivér Závoti, Kenigen Manikion, Bálint Budavári, Angela Jedlovszky Hajdu

**Affiliations:** 1 Department of Surgery, Transplantation and Gastroenterology, Semmelweis University, 1082 Budapest, Hungary; 2 Laboratory of Nanochemistry, Department of Biophysics and Radiation Biology, Semmelweis University, 1085 Budapest, Hungary

**Keywords:** poly(vinyl-alcohol), gel-fibres, hydrogel, electrospinning, surgical

## Abstract

Poly(vinyl-alcohol) hydrogels have already been successfully utilised as drug carrier systems and tissue engineering scaffolds. However, lacking mechanical strength and suturability hinders any prospects for clinical and surgical applications. The objective of this work was to fabricate mechanically robust PVA membranes, which could also withstand surgical manipulation and suturing. Electrospun membranes and control hydrogels were produced with 61 kDa PVA. Using a high-speed rotating cylindrical collector, we achieved fibre alignment (fibre diameter: 300 ± 50 nm). Subsequently, we created multilayered samples with different orientations to achieve multidirectional reinforcement. Finally, utilising glutaraldehyde as a cross-linker, we created insoluble fibrous-hydrogel membranes. Mechanical studies were performed, confirming a fourfold increase in the specific loading capacities (from 0.21 to 0.84 Nm^2^/g) in the case of the monolayer samples. The multilayered membranes exhibited increased resistance from both horizontal and vertical directions, which varies according to the specific arrangement. Finally, the cross-linked fibrous hydrogel samples not only exhibited specific loading capacities significantly higher than their counterpart bulk hydrogels but successfully withstood suturing. Although cross-linking optimisation and animal experiments are required, these membranes have great prospects as alternatives to current surgical meshes, while the methodology could also be applied in other systems as well.

## 1. Introduction

Whilst the development of complex materials is taking the spotlight in current biomedical device research, basic problems with simple solutions are neglected. One area of exceptional and rapid technological development is surgery. Currently, one of the most common issues, regardless of specialisation (general, orthopaedic, or vascular surgery), is postoperative adhesion formation [1,2]. After any procedure, even a minimally invasive one, inflammation, granulation, and scar tissue formation are inevitable [3,4]. This issue is especially prominent in general surgery and specifically the treatment of abdominal hernias [5,6].

A hernia is defined as a protrusion of an internal organ through the cavity it normally resides in [7,8,9]. Hernias most commonly occur in the abdominal region where visceral tissues, typically intestines, due to several causes, can eventually bulge through the abdominal wall. Hernia incidence is quite high; in the USA alone, one million surgeries are performed annually [7,10]. The only definitive treatment is surgery. According to current guidelines, laparoscopic hernioplasty is the first choice involving the implantation of a surgical mesh to support the regeneration of the abdominal wall and prevent recurrence [11,12]. However, the currently utilised synthetic (mostly polypropylene-based), dry, non-absorbable meshes cause adhesions that can lead to severe post-operative complications, including pain, foreign body reactions, mesh migration, and even intestinal perforation [6,13]. Surgical meshes are also utilised for pelvic disorders, for example, uterine or rectal prolapses. Due to the same cause, the same issues (adhesions, uterine-vaginal fistula formation, or intestinal erosion) can also develop in the same manner as hernia surgeries [6,14]. Thus, the search for alternative options began.

One of the most prominent polymers examined in current biomaterial research is poly (vinyl-alcohol) (PVA) [15,16], a synthetic, water-soluble, thermoplastic polymer that is nontoxic exhibits biocompatibility and, under certain conditions, biodegradability [17]. PVA is known for several successful biomedical applications, including drug capsule components, contact lenses, drug carrier systems, and others [18,19,20,21,22,23]. PVA has been investigated before as a potential hernia mesh [9,16] due to its favourable anti-adhesive properties, but unfortunately, due to its poor mechanical performance, it has been put aside while researchers concentrated on other applications.

In this regard, a mechanically enhanced hydrogel could, in the future, combine anti-adhesive and drug-carrier properties serving as an additional layer or a stand-alone hernia mesh. Several methods [21,24] can be used to create such meshes or membranes; however, none is simpler and more versatile than electrospinning. The basic setup only requires a polymer solution, a syringe pump, and a high-voltage power supply along with a grounded collector. From this point onwards, the possibilities for modification are endless. Multi-spinneret, needleless or coaxial electrospinning are just a few examples of how adaptable this technique is [25,26,27,28,29]. Additionally, the electrospinning of PVA has a critical advantage compared to other polymers.

By being a water-soluble polymer, fibre formation can be achieved by using water instead of more volatile and hazardous solvents (e.g., Chloroform, Dichloromethane, Tetrahydrofuran, etc.), a characteristic shared by only a few other synthetic polymers (e.g., polyacrylamide, polyethylene, polyvinylpyrrolidone) [30]. PVA-based materials are also bioabsorbable under physiological conditions. Bioabsorbance highly depends on the molecular weight of PVA. If the molecular weight falls under 10,000 g/mol, it can be excreted through the kidneys; if not, it is eliminated through the gastrointestinal tract [31,32,33]. In some cases, this can be beneficial (i.e., drug carrier systems) for other applications however, (i.e., surgical implants), cross-linking of the system is essential to prevent this early and unwanted dissolution of the system.

Cross-linking can be achieved either physically (e.g., heat treatment, freeze-thawing, radiation mediated) or chemically (i.e., the addition of a cross-linker). Different physical and chemical cross-linking methods have been examined in the past [34,35,36]. The two methodologies, as expected, have both advantages and disadvantages. Physically cross-linked systems using heat treatments, freeze-thawing, or even UV radiation have the advantage of not using cross-linking agents, which as chemicals, could potentially be toxic or cause other unwanted side effects [37]. While these methods do indeed successfully induce cross-links in a PVA system, they are more suitable for bulk hydrogel formation as physical cross-linking would presumably alter the fibrous microstructure of electrospun meshes, either deforming the fibres or melting them, forming a uniform amorphous layer thus eliminating their main advantage, suturability. On the contrary, chemical cross-linking requires chemical agents, which could potentially decrease the biocompatibility of the system, yet microstructure alteration does not typically happen (at least to the same extent as physical methods) [37,38,39]. Important to note is that either method can be optimised; however, in this work we are focusing on the physical reinforcement of the membranes and less on the optimisation of the chemical cross-linking.

Mechanical reinforcement can be physical or chemical. Fibre alignment, and incorporation of additional components, for example, 3D printed fibres, heat treatment, etc., can be categorised as physical reinforcement methods, while chemical reinforcement is typically a cross-linking process or chemical agent addition [23,40,41,42]. One method does not necessarily exclude the other. The presented work focuses on a physical-based reinforcement (fibre alignment and multilayer compression) being the simplest of the two, as it does not significantly alter the chemical composition of the membrane, retains the microstructure but, more importantly, does not limit the possibility of a potential functionalisation in the future (addition of drugs or active agents).

As aforementioned, PVA-based hydrogels have several favourable attributes (non-toxic, non-carcinogenic, biocompatibility, ease of processing, etc.) [23,43,44,45]; however, they lack mechanical robustness. The objective of this work was to fabricate mechanically enhanced, suturable hydrogel PVA membranes that could be utilised for clinical or surgical applications. A lower molecular PVA was chosen as the lower the molecular weight, the easier the biodegradation.

## 2. Materials & Methods

### 2.1. Materials and Reagents

Poly(vinyl-alcohol) (Mowiol 10–98, Mw: 61 kDa, Sigma-Aldrich, St. Louis, Missouri, USA), Glutaraldehyde (25%, Merck, Darmstadt, Germany), Hydrochloric Acid (37%, Reanal Labor, Késmárk, Hungary), Dimethyl sulfoxide (reagent grade ≥99.5%, dehydrated max. 0.03% H₂O, AnalaR NORMAPUR^®^, VWR Chemicals BDH^®^, VWR International, Radnor, PA, USA), Ultra-purified water (Zineer Power I Water Purification System), Poly(glycolic acid), multifilament suture, USP 0/EP 3.5 (Atramat Mexico City, Mexico.)

### 2.2. Preparation of Poly(vinyl-alcohol) Solutions

Briefly, 61 kDa molecular weight PVA powder was mixed with ultra-purified water and then stirred at 120 rpm at 160 °C for approximately 15 min. When the polymer granules dissolved, the heat was turned off, and the stirring continued until the solution reached room temperature. PVA solutions at three different concentrations (5, 10, and 15 *w*/*w*%) were prepared. Polymer concentrations were verified by gravimetric analysis. Glutaraldehye was added to the mixture as a cross-linking agent at different cross-linking degrees (Cross-linking degree: 25, 12.5, 6.25) according to a previously documented method [16]. Additional information can be found in the Supplementary Materials (Section S1).

### 2.3. Electrospinning

**Figure 1:**
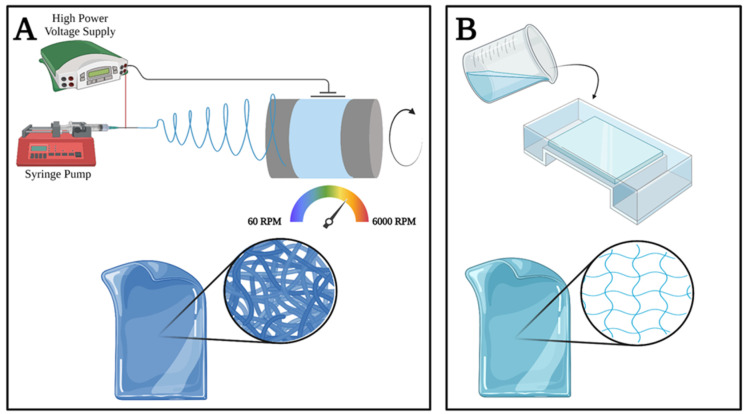
Preparation of poly(vinyl-alcohol) electrospun membranes (**A**) and bulk hydrogels (**B**).

Preliminary samples were fabricated using an immobile, flat collector. With the following parameters: 18 G needle, 0.8 mL/h flow rate, 15 cm needle-collector distance,16 kV voltage, room temperature, and humidity (25 ± 2 °C, 28 ± 5%). Subsequently, meshes were fabricated by utilising a rotating cylindrical collector. Meshes were fabricated at different collector speeds examined, ranging from 60 to 6000 RPM (Figure 1A).

### 2.4. Hydrogel Preparation

PVA hydrogels were created using 15 *w*/*w*% solutions according to the freeze-thawing method and low-temperature crystallisation methods presented in the works of Tomoyo Sakaguchi et al. [46]. Hydrogels were cross-linked in an analogous manner to the electrospun membranes (Figure 1B).

### 2.5. Post-Electrospinning Modifications

**Figure 2:**
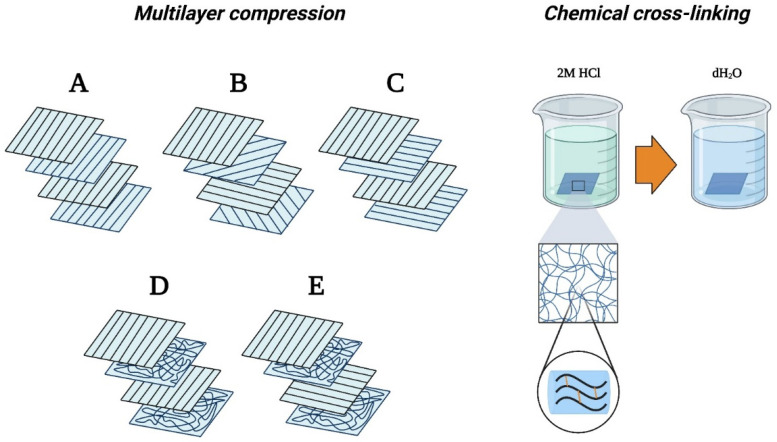
Multilayer compression configurations and cross-linking method. Note: Multilayer Configurations: (**A**) 4  ×  0°, (**B**) 0° + 45° + 90° + 125°, (**C**) 0° + 90° + 0° + 90°, (**D**) 2 × 0° + non-oriented layer (**E**) 0° + non-oriented layer + 90° + non-oriented layer.

After removing the membranes from the collector (Supplementary Figure S1), 10 × 10 cm squares were cut. Four layers with different orientations were placed onto each other. Five different arrangements were designed, as Figure 2 illustrates. Subsequently, the multilayer samples were compressed with 5 tons along their entire surface by a manual hydraulic press. Samples were then immersed in 2 M HCl solution to catalyze the cross-linking reaction. After the samples turned opaque, they were extensively washed in ultrapure water until a neutral pH was reached in the supernatant (Figure 2—Chemical cross-linking).

### 2.6. Physico-Chemical Characterisation

Chemical analysis of the electrospun fibrous meshes was performed using an FTIR spectrophotometer (4700 series type A, JASCO, Tokyo, Japan) equipped with a diamond ATR head (ATR Pro One, JASCO, Japan). All measurements were carried out in a mid-infrared range of wavelength (4000–400 cm^−1^), with 2 cm^−1^ resolutions and 126 total scans. Prior to starting the analysis, background spectra (H_2_O, CO_2_, ATR Head exclusion) were obtained on a clean and dry diamond crystal and were subtracted from the sample spectra. All samples were examined dry (chemically treated samples were extensively dried in a dehydrator beforehand).

To examine the fibre quality and size, scanning electron microscopy was utilised. Small (10 mm^2^) samples were taken from meshes before and after the chemical treatment. Chemically treated samples were frozen and lyophilised before imaging. Images were taken with a JSM 6380LA scanning electron microscope (JEOL, Japan). After securing them on an adaptor with conductive stickers, samples were coated with a thin layer of gold using a JFC-1200 Sputter Coating System (JEOL, Japan). The applied voltage was 15 kV, and micrographs were obtained at 1000×, 2500×, and 5000× magnifications. Average fibre diameter and size distribution were determined by measuring 100 individual fibres. Fibre alignment was automatically calculated by the orientation function of the utilised software. All imaging analysis was performed using Fiji software (Version, 1.53t 24 Open-Source Software).

### 2.7. Mechanical Characterisation

**Figure 3:**
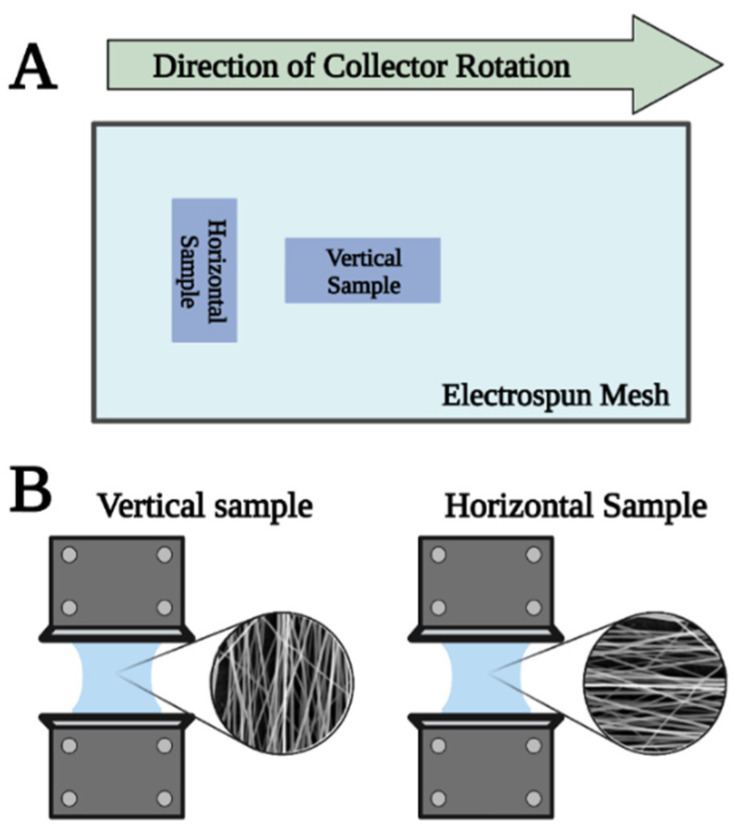
Cutting of samples for mechanical evaluation (**A**) and fibre orientation differences (**B**) in the case of monolayer membranes.

For the assessment of the specific loading capacity, we utilised a 5942 uniaxial tester (Instron, Norwood, MA, USA). Rectangles measuring 2 × 6 cm sized were cut out from the electrospun meshes labeled as vertical and horizontal according to the direction of fibre orientation (Figure 3). Samples were placed between the clamps of the mechanical tester and were pulled at 1 mm/min speed until tearing. In addition to their small thickness, electropsun meshes are considered soft materials. Thus, mechanical performance was evalutated by calculating specific loading capacity considering the samples’ surface density.

Specific loading capacity (Nm2g)=Maximum sustained load (N)Surface density (gm2)

where

$$Surface\;density\left(\frac{g}{m^{2}}\right)=\frac{Mass\;\left(g\right)}{Surface\;area\;\left(m^{2}\right)}$$

Preliminary tests were performed examining the effect of fibre alignment (500, 2000, 4000, and 6000 rpm). Subsequently, multilayer meshes were evaluated. Following the cross-linking procedure, we evaluated membranes under physiological saline.

**Figure 4:**
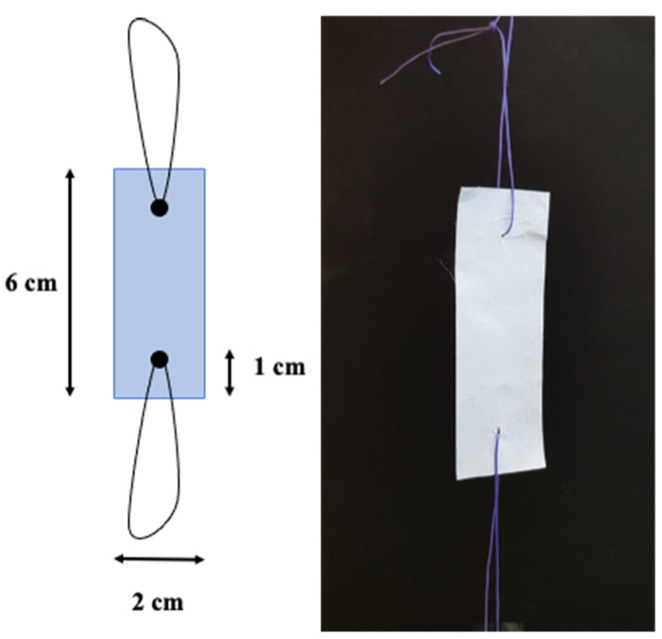
Sutured model for mechanical assessment.

To examine suturability, samples were sutured on both ends via a simple interrupted suture before the measurement (Figure 4).

### 2.8. Statistical Analysis

Parametric independent *t*-tests were performed to examine the significance of fibre diameters and specific loading capacity values among different samples. Pearson test was also performed for the fibre diameter-specific loading capacity correlation. For the analysis, STATISTICA 10 software (TIBCO Software Inc., Palo Alto, CA, USA) was used.

## 3. Results

### 3.1. Poly(vinyl-alcohol) Electrospinning

**Figure 5:**
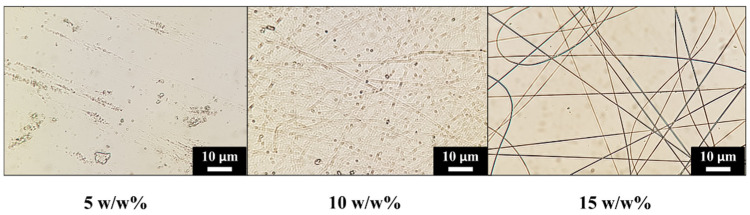
Fibre formation confirmation with light microscopy.

**Figure 6:**
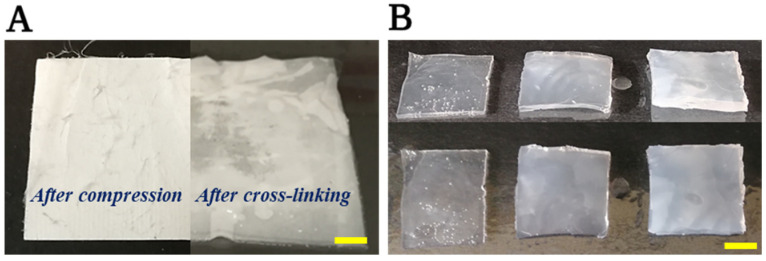
Electrospun PVA membrane (**A**) before (left) and after (right) chemical treatment, Hydrogels (**B**) prepared by (low-temperature crystallisation method (left), freezing-and-thawing method (middle), hot-pressing method (right). Note: Scale bar at 0.5 cm.

To our knowledge, PVA of 61 kDa has not been used for electrospinning yet by other research groups. After a short preliminary electrospinning session, the specimens were examined under a light microscope. Evidently, 15 *w*/*w*% is the minimal concentration at which PVA fibre formation was possible without aberration on the fibres (Figure 5). Thus, control hydrogel was also prepared at a 15 *w*/*w*% concentration. After electrospinning, meshes are initially fleecy and white (Figure 6A—left). After the chemical treatment, meshes become elastic and opaque (Figure 6A—right). In contrast, PVA hydrogels are typically fragile, while their transparency depends on the production method (Figure 6B).

### 3.2. Physico-Chemical Characterisation

**Figure 7:**
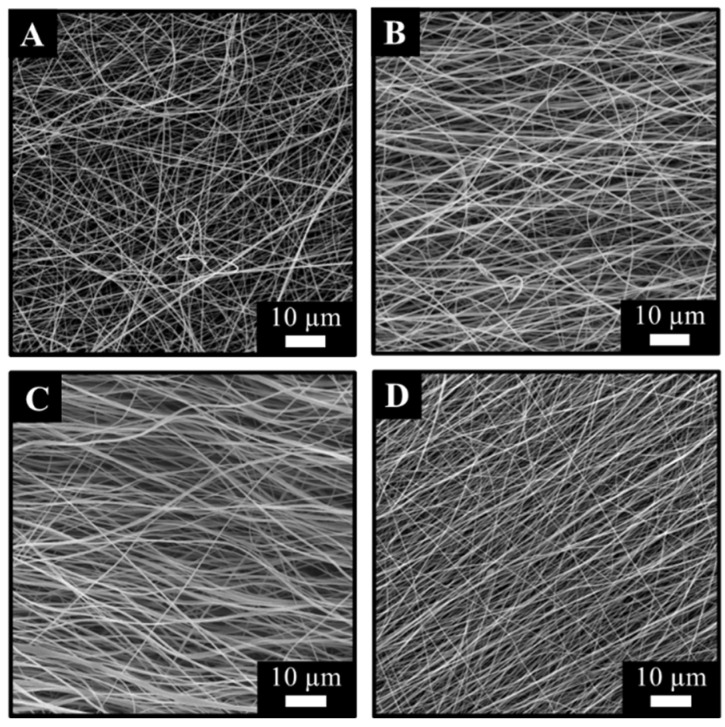
Scanning electron microscopy of electrospun membranes fabricated at (**A**) 500 rpm, (**B**) 2000 rpm, (**C**) 4000 rpm, and (**D**) 6000 rpm collector speeds.

**Figure 8:**
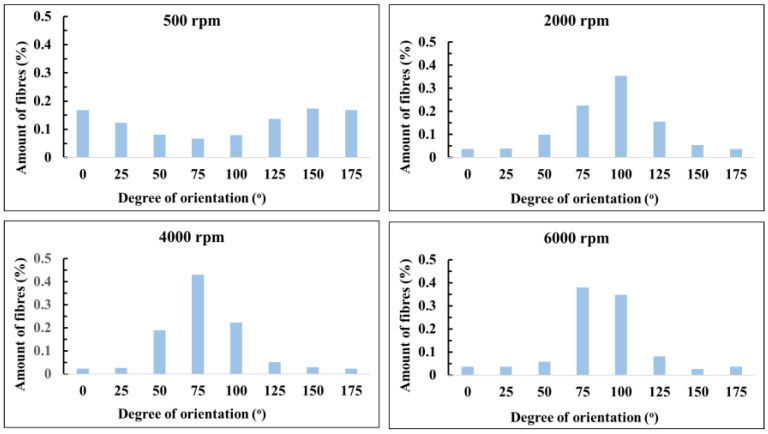
Orientation distribution of aligned meshes.

All membranes were successfully fabricated without fibre aberrations such as beads, bubbles, or other artifacts. At 500 rpm, the fibres orient equally in each direction, looking remarkably similar to the meshes produced on the static immobile collector (Supplementary Figure S2) with no apparent difference in alignment. With higher collector speeds, however, the alignment increases (Figure 7 and Figure 8). At 2000 rpm, approximately 80% of the fibres are aligned within a 50-degree range (Figure 8). This slightly increases with the 4000 rpm samples (Figure 8). The 6000 rpm membranes are the most oriented, where most of the fibres (approximately 80%) are found within a 25-degree range (Figure 8). Evidently, increasing the collector speed leads to greater fibre orientation, albeit non-aligned fibres will always be present to some degree.

Having calculated the average fibre diameter values for each collector speed setting (Table 1), it was apparent that the higher the collector speed, the smaller the average fibre diameter. At 500 rpm, typically, fibres of approximately 500 nm diameter (480 ± 60 nm) could often be observed. The 2000 rpm and 4000 rpm samples looked remarkably similar on the scanning electron microscopy images, and just like in the case of the orientation, no significant difference (“unpaire” *t*-test) could be detected in fibre diameter values. At 6000 rpm, however, the average diameter decreased by more than 50% compared to samples produced by the immobile collector (*p* < 0.001) (Table 1).

**Figure 9:**
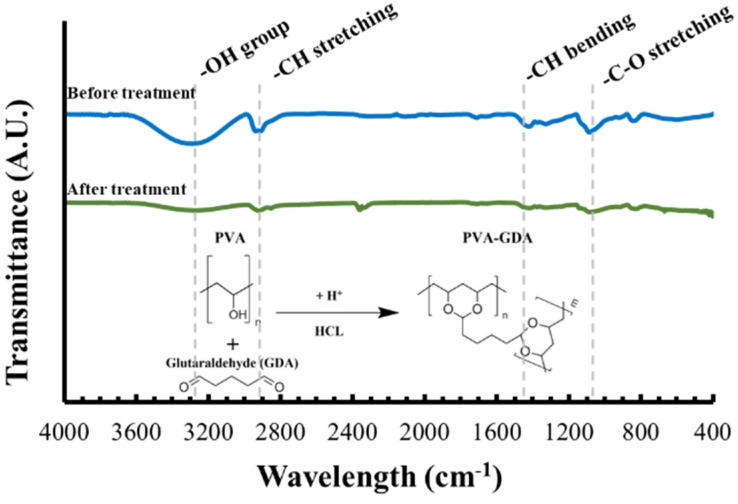
Chemical analysis and cross-linking confirmation.

Chemical cross-linking starts directly after glutaraldehyde (GDA) is added to the polymer solution before electrospinning; nevertheless, its full effect only takes place only after the samples are immersed in an acidic solution (in this case, 2 M HCl). Normally, as PVA is water-soluble, non-treated PVA meshes would dissolve in water, but since the chemically treated samples persisted under the storage for more than a month, with no physically visible structural changes or material loss, we could assume the completion of cross-links was successful. This was further confirmed by the ATR-FTIR spectroscopy measurements (Figure 9). More specifically, examining PVA membranes spectra, the -OH peak at 3303 cm^−1^ decreases after the chemical treatment as the GDA cross-linking is successful.

**Figure 10:**
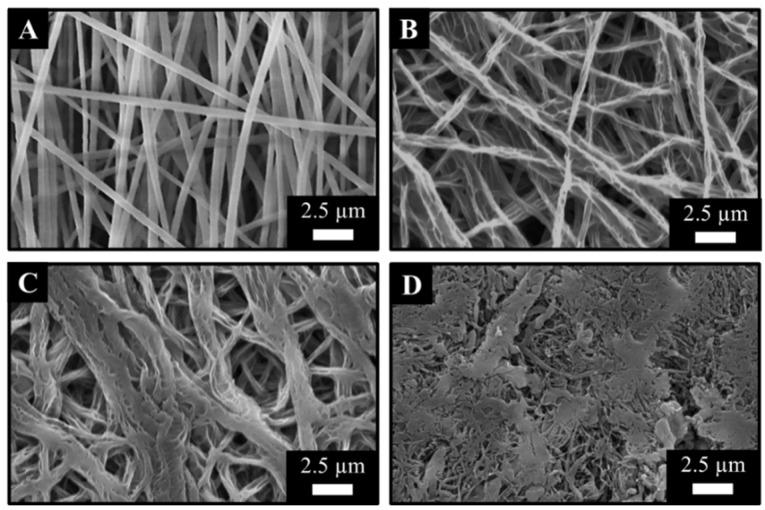
Scanning electron microscopy of hydrogel fibre membranes before (**A**) and after chemical treatment with 25 (**B**), 12.5 (**C**), and 6.25 (**D**) cross-linking degrees.

Scanning electron microscopy was used to assess any microstructure differences after the cross-linking (Figure 10). As expected, cross-linking results in the absorption of the surrounding liquid and the creation of hydrogel fibres. The average fibre diameter is increased, measuring 580 ± 80 nm (cross-linking degree: 25). It is important to clarify that cross-lining occurs between two polymer chains that are located within a single polymer fibre. According to the cross-linking degree, however, at some point, fibres merge due to cross-links between polymer chains within different fibres (Figure 10C). By further increasing the cross-linker concentration, fibres seem to merge into a single layer, which contains areas where fibres have not completely merged yet (Figure 10D). For the mechanical assessment, samples with a cross-linking density of 25 were used, being the most reproducible in terms of microstructure.

### 3.3. Mechanical Evaluation

#### 3.3.1. Monolayer Membranes

**Figure 11:**
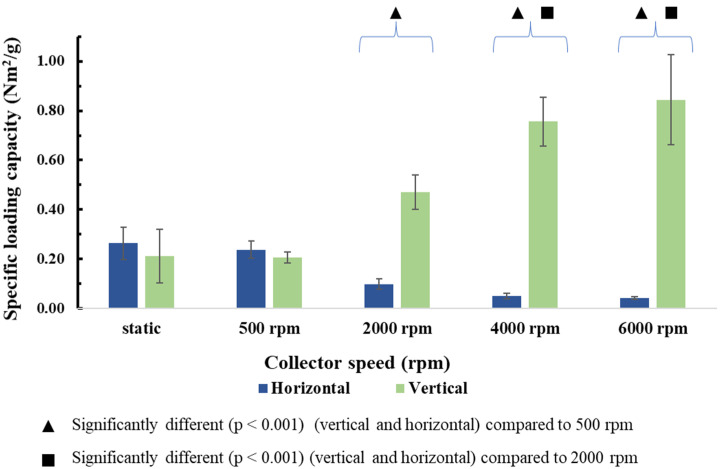
Specific loading capacities of monolayer membranes.

Representative stress–strain curves of these membranes (Supplementary Figure S3) resemble typical electrospun system curves found abundantly in the relevant literature [16,40]. At 500 rpm, mechanical performance was not significantly different from samples produced with the static immobile collector. However, as alignment increases at 2000 rpm and above, so does the specific loading capacity (Figure 11). In contrast, performance on a 90° degree angle (horizontal samples) decreases. Similar findings were documented by other electrospinning works as well [47].

The significant difference in Figure 11 was calculated using unpaired tests. As the fibre diameter cannot be excluded in this setting, the Pearson correlation test was used to examine the average fibre diameter (affected by collector speed) and specific loading capacity relationship. According to the test, a strong negative correlation is observed (Pearson coefficient: −0.775).

#### 3.3.2. Multilayer Membranes

**Figure 12:**
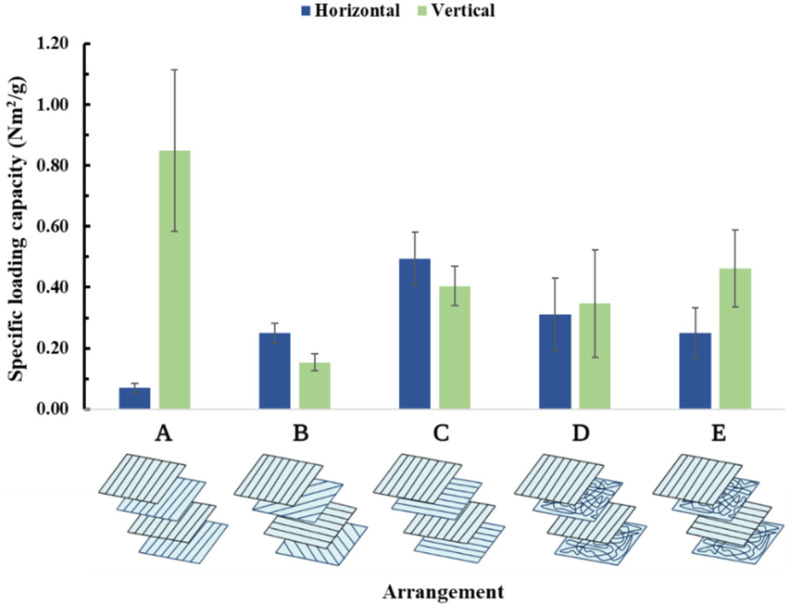
Specific loading capacities of multilayer membranes.

Due to the previous findings, multilayer meshes were prepared using samples prepared at 6000 rpm collector speeds (Figure 2). As expected, multilayered meshes were more resistant to the pulling forces than the monolayered samples. Although higher forces were necessary to tear the samples, the specific loading capacities decreased in the vertical direction (except, of course, in case ‘A’) compared to their monolayered counterparts, and multilayer membranes became more resistant in the horizontal direction (Figure 12). Out of all the arrangements, case C, where two vertical and two horizontal layers were used, seems to be the most reliable, with the best overall performance and least variance. Additional figures (Supplementary Figures S4 and S5) and the statistical analysis can be found in the Supplementary Materials.

**Figure 13:**
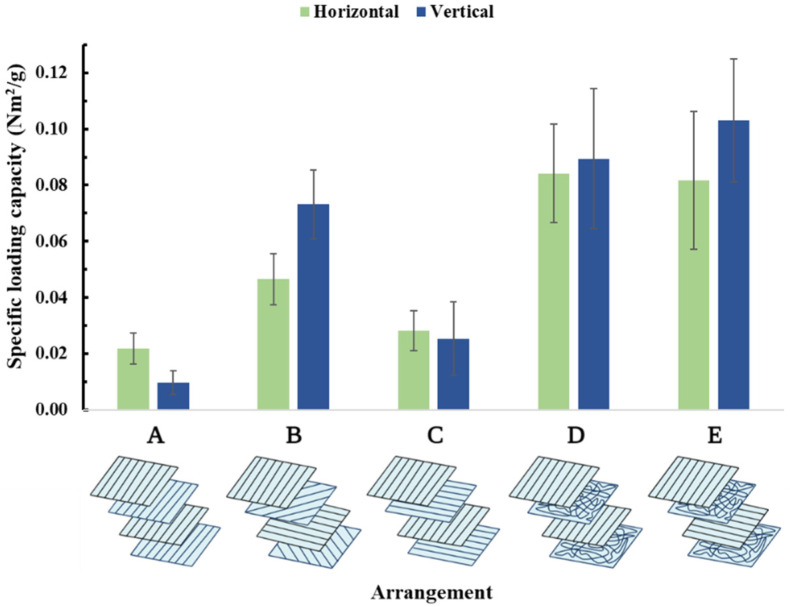
Specific loading capacities of the sutured multilayer membranes.

Since the main objective of the membranes is going to be clinical and surgical application, examination of suture retention is paramount. Thus, multilayer compressed membranes were sutured and re-assessed. According to our results, suturing not only creates a defect in the membranes, but as the force is only exerted along the suture material, the mechanical tension is huge. Taking a closer look at the stress–strain curves, the tearing of the individual layers can be seen (Supplementary Figure S5). In general, the specific loading capacity values were an order of magnitude less than the values obtained in the simple mechanical test, as the exerted force is divided by the diameter of the suture lines. Case ‘A’ proved to be the least resistant to vertical directional pulling. Although cases ‘B’ and ‘C’ had symmetrical and equal mesh arrangements, cases ‘D’ and ‘E’ turned out to be the strongest due to the additional “suture resistance” provided by the randomly oriented layers (Figure 13). Additional figures (Supplementary Figures S6 and S7) and the statistical analysis can be found in the Supplementary Materials.

#### 3.3.3. Hydrogel Membranes

The previous assessments proved how multilayer compression is an effective method to reinforce membranes in multiple directions. For that reason, the hydrogels were made from compressed meshes to determine the best performance. Similarly, the five arrangements were used as with the dry samples. For the mechanical assessment, the 25 cross-linking degree was chosen, as during preliminary investigations using scanning electron microscopy evaluation, they proved the most reproducible in terms of microstructure. Before and during the procedure, all the samples were kept under physiological saline (Supplementary Figure S8). After the measurements, gravimetric analysis was also performed in the case of a few samples, which confirmed that the PVA content of the hydrogels was indeed 15 *w*/*w*%.

**Figure 14:**
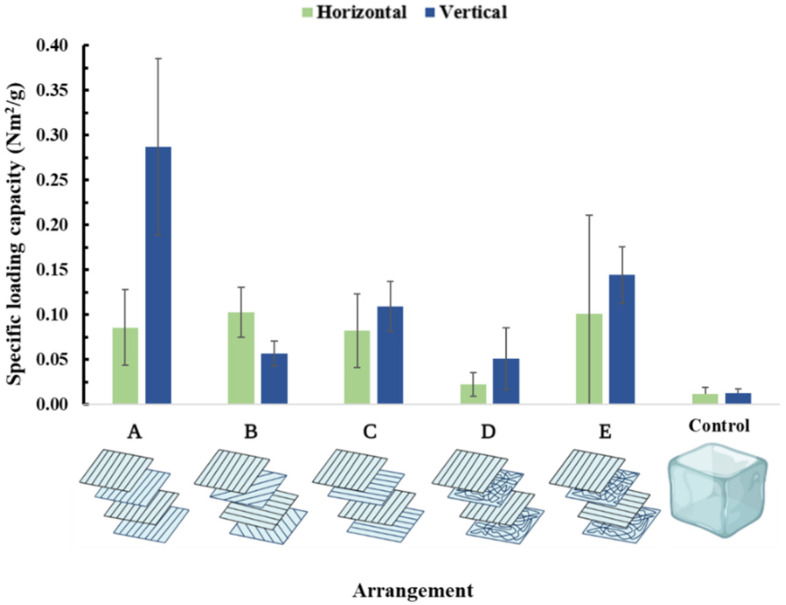
Specific loading capacities of the hydrogel membranes.

According to our results, the chemical treatment affected not only the microscopic structure of the meshes but also their mechanical properties. Membranes performed worse than their dry counterparts (Figure 14). However, when compared to the bulk hydrogel membranes, the fibrous hydrogel membranes outperform them significantly (*p* < 0.001) in almost every case (Figure 14, Supplementary Figure S9).

**Figure 15:**
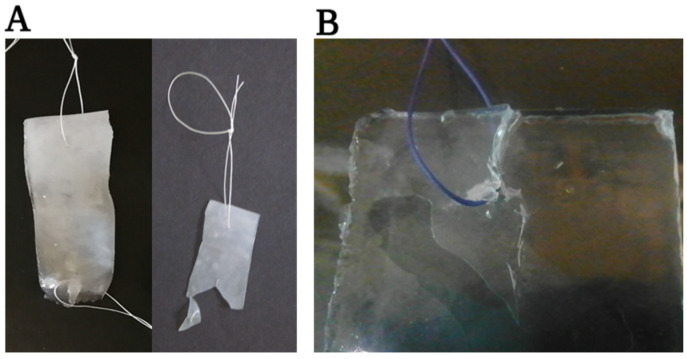
Suturability of poly(vinyl alcohol) electrospun membranes before and after uniaxial measurement (**A**) and bulk hydrogels (**B**).

Furthermore, when investigating suturability, it was evident that while the electrospun membranes can be easily manipulated and sutured, bulk hydrogels fall short. Unfortunately, during their handling, the suturing process simply cuts through the bulk hydrogel even with minimal effort, making suturing impossible. In contrast, the electrospun membranes not only withstood the simple suturing (Figure 15) but can also withstand complex suture lines and can be assessed by the mechanical tester (Supplementary Figure S10).

## 4. Discussion

Biomedical material research is more popular than ever as the demand for novel biomaterials is still high in the medical field. The aim of this work was to develop electrospun fibrous membranes from poly(vinyl-alcohol) with the potential to be used in clinical or surgical applications. From a biocompatibility point of view, PVA is already regarded as a suitable material for humans, and it can be found in several market-available products, for example, as drug capsules [18,19,20,21,22,23]. From a surgical point of view, poly(vinyl alcohol) exhibits anti-adhesive properties, which could limit the unregulated development of granulation tissue and postoperative adhesions that lead to complications [16].

In this regard, PVA hydrogels have been the focus of many researchers as they provide an excellent medium for drug delivery while also being non-toxic, easy to manufacture, and low-cost [18,19,20,21,22,23]. However, due to their poor mechanical performance, further application possibilities are limited. To circumvent this issue, we investigated electrospun PVA membranes and aimed to enhance them, increasing their mechanical performance and making them resistant to surgical manipulation. In addition, if successful, the membranes could also be functionalised in the future as drug-carrying surgical meshes.

It is worth discussing the molecular weight of poly(vinyl alcohol). It is a well-known fact that increasing the molecular weight will result in more robust electrospun fibres and membranes. According to our best knowledge, we are the first to successfully produce hydrogel fibre membranes using such a low molecular weight. A PVA of this low molecular weight was chosen as it is approved by the FDA (U.S. Food and Drug Administration), so it has already been proven safe for human use. In addition, the lower the molecular weight, the faster the degradation of the mesh in the body [33]. An ideal membrane should degrade over time to allow the complete recovery and fusion of tissues. A rapidly degrading membrane promotes the creation of the granulation tissue and thus the healing of the abdominal wall; however, if degraded too quickly however, it will not support the abdominal wall, resulting in hernia recurrence [48]. Therefore, if effective, the method can be utilised with different molecular weight PVA according to the requirements of the specific application.

According to our preliminary experiments, 15 *w*/*w*% solutions can be reliably used to fabricate membranes composed only of nanofibres. Using lower concentration may be possible with the additions of an ionic component (e.g., NaCl) to increase the solution’s conductivity or by using binary solvents (e.g., H_2_O/DMSO) to also increase the evaporation rate of the solutions [49] however; such investigations extend over the focus of this work. Higher concentration PVA solutions were also briefly investigated; however, they were considerably more difficult to prepare, requiring more adjustments, thus making the process er to reproduce. In addition, higher concentration results in higher viscosity which can hinder the electrospinning process due to technical difficulties (flow rate issues).

By applying a cylindrical, rotating collector, not only were we able to fabricate aligned fibres, but the electrospinning sessions also became faster when for example, two syringes were applied in opposite directions. As the two syringes were positioned a little shifted to the sides to avoid interference, more consistent and approximately equally thick meshes can be produced. This helped the manual separation of the specimens from the collector and minimised the loss of material, which was a recurring problem with the static collector (Supplementary Figure S1).

The microstructure of the membranes was examined before and after the post-electrospinning treatments. Throughout the fabrication process, the fibrous microstructure of the membranes remained undamaged. By increasing the collector speed above 500 rpm, fibre alignment started to appear. Further increasing the collector speed also decreased the average fibre diameter. The final average diameters were increased after the chemical treatment due to the absorption of the surrounding liquid and hydrogel fibre formation. Fibre alignment also had evident effects on the mechanical performance of the membranes as specific loading capacity (monolayer samples) increased fourfold (from 0.21 to 0.84 Nm^2^/g). While the effect of a decrease in fibre diameter cannot be excluded in this setting, it is clear that alignment significantly increases mechanical performance along the direction of the orientation.

Mechanical strength can be described by various models. Young’s modulus is derived by dividing the exerted tensile stress (Force/Area) by the extensional strain or deformation (ΔL/L_O_) at the initial linear elastic portion of the stress–strain curve. Additionally, ultimate tensile strength could also be expressed by dividing the maximal exerted load by the surface area of the sample (σ = Force/Area). These models present two difficulties. Firstly, these membranes, being polymer based, are not perfectly in line with a Hookean linear model stress–strain behavior. Alternatively, Neo-Hookean or Mooney–Rivlin models can be used, albeit their accuracy has been questioned before [50,51]. Secondly, the samples are thin and soft, and thus, their thickness can not be accurately measured by digital calipers. In this regard, surface area (Surface Area = Width x Thickness) cannot be calculated. To solve this issue, surface density and specific loading capacity were used as performed previously by our research group. Specific loading capacity takes mass into account; thus, the mechanical performance of such light samples of varying thickness and mass are more objectively compared.

In the case of biomedical materials intended for surgical applications, multidirectional resistance is crucial. To enhance the membranes in this manner, we investigated multilayer compression of differently aligned arrangements. Analysing the specific loading capacity of the four-layered compositions:

- When four samples are compressed parallelly, there is no notable difference in the specific loading capacity values neither from vertical nor from horizontal pulling in comparison with the monolayered samples, as the increased maximum load is divided by the increased mass; thus, the final specific loading capacity is the almost the same.
- The samples exhibited a decrease in specific loading capacity in the vertical direction and an increase in the horizontal one. The concept of this arrangement was that force would be equally exerted from all directions; however, to the nature of the measurement (cutting vertical and horizontal samples), this effect is overshadowed. Utilising a bi-axial mechanical tester would provide further insight; however, such equipment was not available.
- This cross arrangement provides horizontal and vertical reinforcement, which is accurately detected by the mechanical tester. Of course, compared to an all-vertical arrangement, the vertical direction suffers, but in exchange, the horizontal direction is compensated. For example, compared with the meshes made in case ‘B’, significantly higher values could be observed with this setup because relatively more fibres were facing in the two main pulling directions.
- Similar behaviour to case C. The randomly oriented meshes between the oriented layers added to the overall resistance of the arrangement, which could be detected on the specific loading capacity values sitting somewhere between the values measured in cases ‘B’ and ‘C’.
- Similar behaviour to cases C and D. In this setup, a slight difference was observed in the two specific loading capacity values. In comparison to the vertical samples, there were no oriented meshes supporting the horizontal directional pulling, and as a result, almost half the specific loading capacity was documented. Only the randomly oriented meshes were adding support to the horizontal samples; that is the reason these values are still significantly higher than in case ‘A’, where the samples had no horizontal reinforcing.

Since surgical meshes must be fixated, it is critical to examine their behaviour and mechanical resistance after suturing. In addition, further insights regarding the layer arrangement can be gained after examining the sutured model counterparts. Analysing the specific loading capacity values of the multilayered, sutured meshes:

- Unlike the previous measurement, this arrangement performed the worst in both vertical and horizontal directions. This can be explained since the suture slips and passes through the fibres (vertical direction) or finds not have enough resistance (horizontal direction).
- In this case, a slightly higher vertical value compared to case ‘A’ due to the two oblique individual layers.
- Interestingly this arrangement underperformed compared to cases ‘B, C, and D‘. It seems that oblique layers provide more resistance against suture than horizontal layers. This suggests that although in laboratory settings, this arrangement would be the strongest and all-around most trustworthy, in a real-life setting, this would not be true.
- In this arrangement, the randomly oriented layers provided resistance against the sutures. Similar values were assessed; this is also a symmetrical composition.
- The highest values from vertical directional pulling were measured in this case. This is presumably due to the stabilising effect of the randomly oriented meshes, as the other specimens mentioned earlier showed that the fibres parallelly aligned with the pulling do not strengthen the samples but rather make it easier for the stitch to cut through the fibres. However, here no significant difference was calculated in the specific loading capacity values in comparison to case ‘D’.

To summarise, case ‘A’ proved to be the strongest from vertical directional pulling, with the specific loading capacity values measured remarkably similar to the monolayered samples. If we consider the two main pulling directions, case ‘C’ is approximately equally strong from either vertical or horizontal direction while also exhibiting the least variance among samples. Although the specific loading capacity values were the lowest in case ‘B’, its true potential can only be fully explored with a bi-axial mechanical tester. Cases ‘D’ and ‘E’can be interpreted as a blend of cases ‘B’ and ‘C’ as this composition has fewer fibres facing in the main pulling directions, but it is still supported from all directions.

Furthermore, this assessment shows that a surgical mesh is just as strong as its weakest point, which is the place of the suture. The stitches were disuniting the fibres and damaged the structural integrity of the samples; thus, overall lower specific capacities were documented. Suturing further complicates matters as the symmetrical arrangement ‘C’ suffers the most. Suturing can either cut through or slips through the fibres. In this regard, random layers seemingly provide the best resistance, as exhibited by arrangments ‘D’ and ‘E’. While case ‘E’ (suture model) seems better compared to arrangement ‘B,’ it comes with high variance due to the random layers. Thus arrangement ‘D’ seems to be the best option for a real-life scenario combining mechanical strength and suture resistance.

As strong as these membranes are, all would be lost without the addition of a cross-linker which prevents the immediate dissolution of the system. Our research group has previously investigated glutaraldehyde as a cross-linker with positive results. Although the cross-linker is added before the electrospinning, its full effect takes place during the immersion into an acidic environment. Macroscopically, membranes become firstly transparent, and then after thoroughly washing them, they become opaque. These fibrous-hydrogel membranes are elastic and durable. According to the scanning electron microscopy images, increasing the cross-linking density results in cross-linking between polymer chains among different fibers and fibre merging. At a 6.25 cross-linking density, the fibrous microstructure is lost. Thus, the mechanical investigation in this work was performed using the 25 cross-linking degree samples. Optimising cross-linking degree and examining alternative cross-linkers (e.g., citric acid, boric acid) extends over the focus of the current work and will be presented in a future paper.

Considering the specific loading capacity values, the PVA hydrogels are mechanically more durable than the monolayered meshes. In addition, compared to bulk hydrogel prepared at the same concentration (15 *w*/*w*%), they perform significantly better. Compared to the dry multilayer-compressed fibrous hydrogel, samples performed worse while the arrangement exhibited equivalent properties to their dry counterparts. The quantity of glutaraldehyde or an alternative cross-linker can still be optimised in the future. Nevertheless, the primary goal of outperforming bulk hydrogels was achieved, along with samples being resistant to surgical manipulation and suturing.

## 5. Conclusions

To summarise, electrospun membranes were fabricated from a low-molecular-weight poly(vinyl alcohol) of 61 kDa. Utilising mechanically induced fibre alignment and multilayer compression, robust suturable membranes were prepared. Fibre alignment significantly decreases average fibre diameter and increases specific loading capacity. We examined multiple fibre orientation arrangements. According to our results, fibre alignment can be used to strengthen “conventional“ mechanical performance by enhancing mechanical resistance from both vertical and horizontal directions (using aligned layers). Materials should be tested as close as possible to their real-life application. In this regard, randomly aligned layers proved crucial as they provide better resistance against sutures than aligned layers. Using glutaraldehyde as a cross-linker, insoluble fibrous hydrogel membranes were created, which not only outperformed control bulk hydrogels but can withstand manipulation and suturing. PVA is an immensely popular material amongst biomedical materials for several reasons. Due to poor mechanical performance, applicability is limited. This, however, can be overcome by utilising the methods presented in the current work.

## Figures and Tables

**Table 1:** Average diameter of electrospun membranes.

Collector Speed (rpm)	Average Fibre Diameter (nm)
0 rpm	520 ± 100
500 rpm	480 ± 60
2000 rpm	440 ± 70
4000 rpm	440 ± 90
6000 rpm	300 ± 50

## Data Availability

The data presented in this study can be requested from the corresponding author for a reasonable reason.

## Supplementary Materials

The following supporting information can be downloaded at: https://www.mdpi.com/article/10.3390/membranes13010116/s1, Figure S1. Membranes directly after extraction from collector. Figure S2. Scanning electron microscopy of membranes fabricated with the static immobile flat collector. Figure S3. A representative vertical (left) and horizontal (b) stress-strain curves of the monolayer PVA membrane. Figure S4. Multilayer membrane during mechanical assessment. Figure S5. A representative stress-strain curves of a multilayer “C Arrangement” PVA membrane vertical sample (Green arrows: layer tearing). Figure S6. Sutured Multilayer membrane during mechanical assessment. Figure S7. A representative vertical (left) and horizontal (b) stress-strain curves of the sutured “C Arrangement” multilayer PVA membranes (Green arrows: layer tearing). Figure S8. Hydrogel PVA membrane mechanical assessment. Figure S9. Stress-strain curves of an electrospun membrane (vertical sample) and a bulk hydrogel. Figure S10. Suturability of electrospun PVA membranes. Table S1: Explanation of the significance levels calculated using unpaired t-test analysis. Table S2: Analysis of the multilayered vertical samples. Table S3: Analysis of the multilayered horizontal samples. Table S4: Analysis of the multilayered, sutured vertical samples. Table S5: Analysis of the multilayered, sutured horizontal samples.
