# Supplementary material for: Fabrication of Mechanically Enhanced, Suturable, Fibrous Hydrogel Membranes
Source: Membranes (Basel). 2023 Jan 16;13(1):116. doi: 10.3390/membranes13010116 (PMC9867240; doi:10.3390/membranes13010116)
Supplement: Supplementary file 1 [file membranes-13-00116-s001.zip › membranes-2103037-supplementary.pdf]

membranes

MDPI

Supplementary Material

Fabrication of Mechanically Enhanced, Suturable, Fibrous Hydrogel Membranes

Constantinos Voniatis [1,2,*], Olivér Závoti [2], Kenigen Manikion [2], Balint Budavári [2] and Angela Jedlovszky Hajdu [2]

1 Department of Surgery, Transplantation and Gastroenterology, Semmelweis University, 1085 Budapest, Hungary
2 Laboratory of Nanochemistry, Department of Biophysics and Radiation Biology, Semmelweis University, 1085 Budapest, Hungary
* Correspondence: voniatis.konstantinos@med.semmelweis-univ.hu

Citation: Voniatis, C.; Závoti, O.; Manikion, K.; Budavári, B.; Hajdu, A.J. Fabrication of Mechanically Enhanced, Suturable, Fibrous Hydrogel Membranes. *Membranes* **2023**, *13*, 116. https://doi.org/10.3390/membranes13010116

Academic Editor: Rafael Torres-Mendieta

Received: 1 December 2022
Revised: 9 January 2023
Accepted: 10 January 2023
Published: 16 January 2023

Copyright: © 2023 by the authors. Submitted for possible open access publication under the terms and conditions of the Creative Commons Attribution (CC BY) license (https://creativecommons.org/licenses/by/4.0/).

S1. Materials and Methods

<u>Chemical cross-linking</u>

Since PVA is water-soluble, it is essential to cross-link the polymer molecules to prepare a water-insoluble, polymer gel system, which could eventually be implantable to the body. For this purpose, glutaraldehyde (GDA) was mixed into the PVA solutions as a cross-linker. An equal volume of 1 M GDA was added to 15 w/w% PVA solutions to achieve different crosslinking densities. Cross-linking density was calculated as follows:

$$Cross - linking\ Density = \frac{amount\ of\ crosslinks\ (mol)}{total\ polymer\ mass\ (mol)}$$

For example, 4 grams of 15 w/w% PVA solution contain 0.6 grams PVA,
which equals to 1,36 × 10^{-2} mol (calculated by the monomeric weight of PVA, 44 g/mol).
Thus, 4 g × 0.15 = 0.6 g then (0.6 g)/(44 g⁄mol) ≈ 0.0136 mol

In order to cross-link every 25th PVA molecule in the polymer chain:
(0.013 mol)/25=5.44 × 10^{-4} mol and 5.44 × 10^(-4) mol PVA should be in bond.

Since one molecule GDA can bind two PVA monomer molecules:
(5.44 × 10^{-4} mol)/2 = 2.72 × 10^{-4} mol

Thus, 2.72 × 10^{-4} mol GDA is needed.

<u>Post-electrospinning modifications</u>

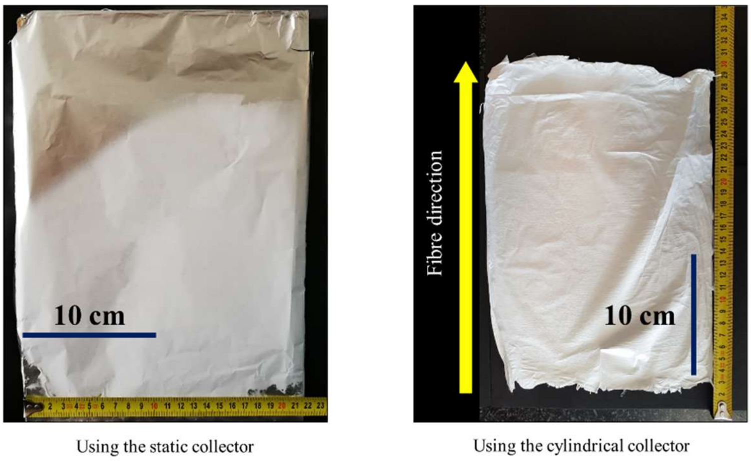

Supplementary Figure S1. Membranes directly after extraction from collector.

S2. Results

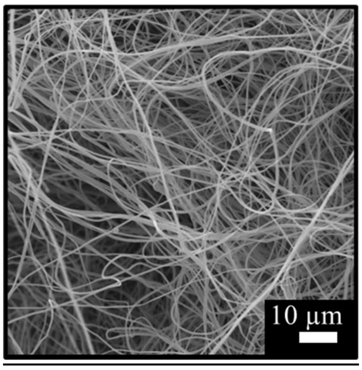

Supplementary Figure S2. Scanning electron microscopy of membranes fabricated with the static immobile flat collector.

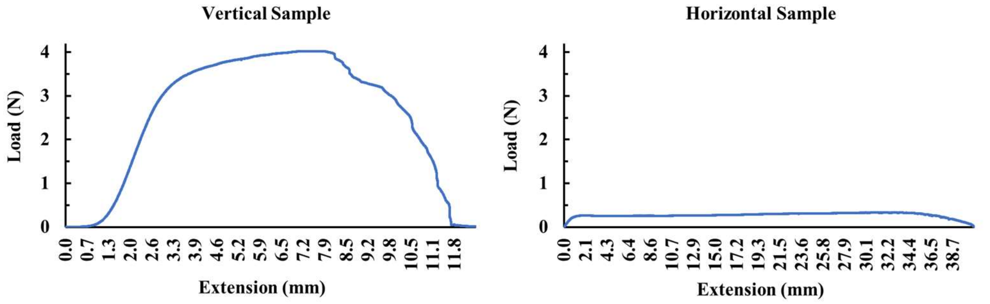

Supplementary Figure S3. A representative vertical (left) and horizontal (b) stress-strain curves of the monolayer PVA membrane.

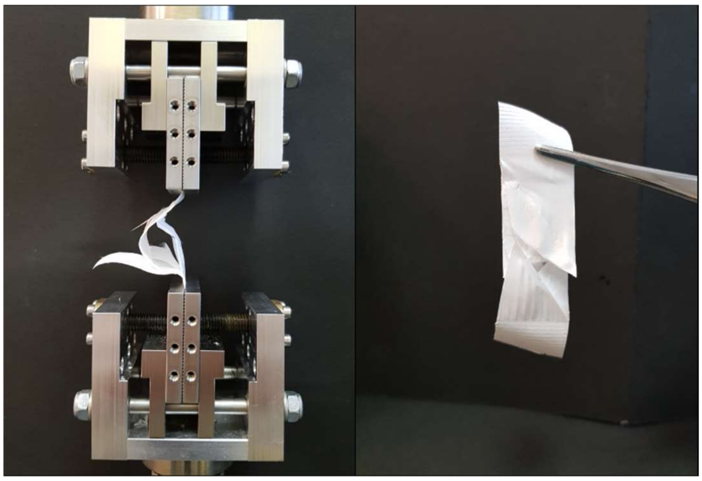

Supplementary Figure S4. Multi-layer membrane during mechanical assessment.

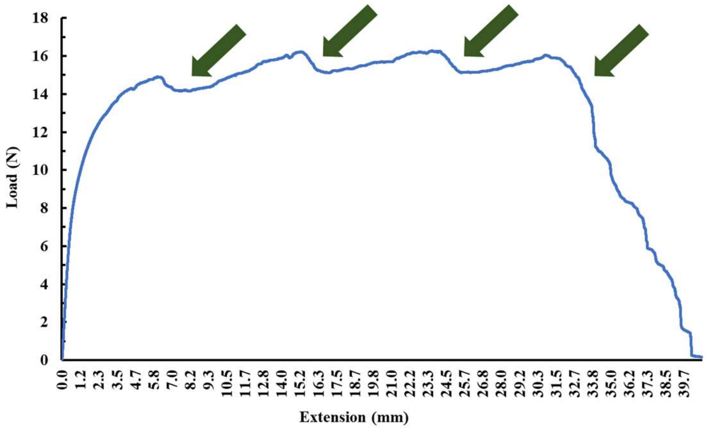

Supplementary Figure S5. A representative stress-strain curves of a multi-layer "C Arrangement" PVA membrane vertical sample (Green arrows: layer tearing).

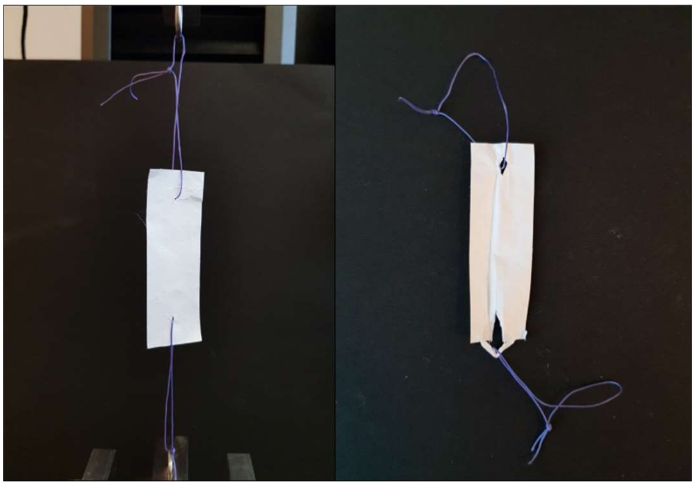

Supplementary Figure S6. Sutured Multi-layer membrane during mechanical assessment.

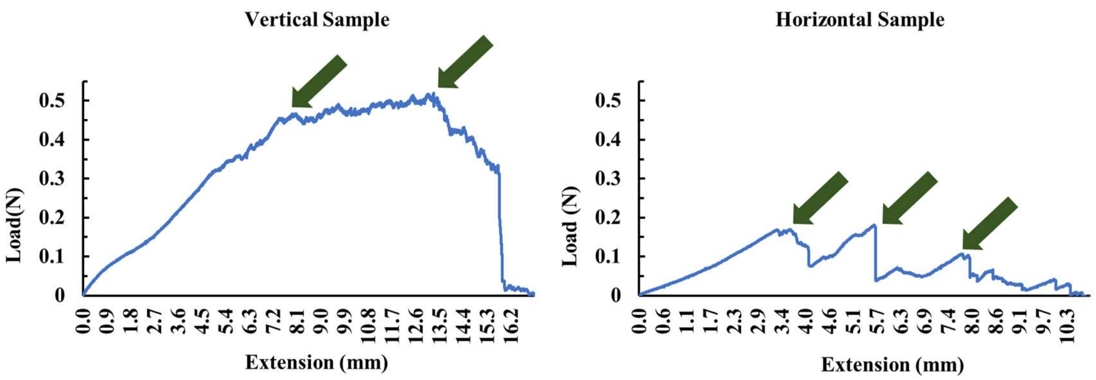

Supplementary Figure S7. A representative vertical (left) and horizontal (b) stress-strain curves of the sutured "C Arrangement" multi-layer PVA membranes (Green arrows: layer tearing).

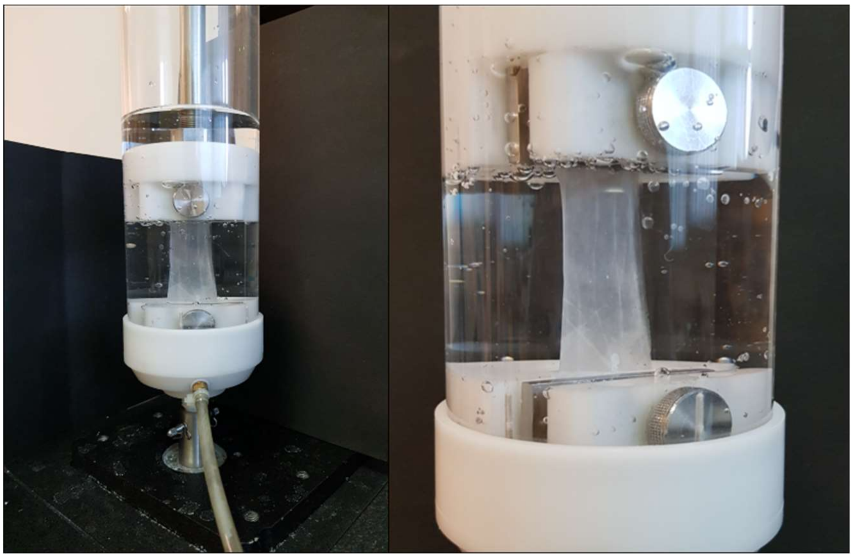

Supplementary Figure S8. Hydrogel PVA membrane mechanical assessment.

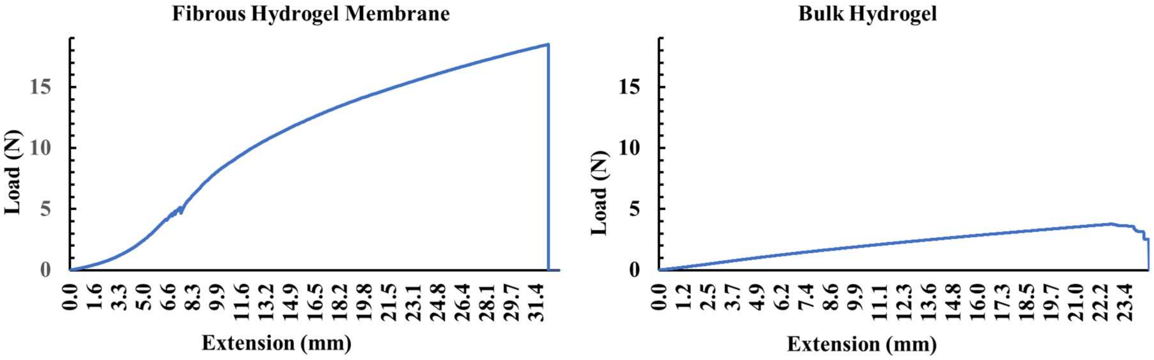

Supplementary Figure S9. Stress-strain curves of an electrospun membrane (vertical sample) and a bulk hydrogel.

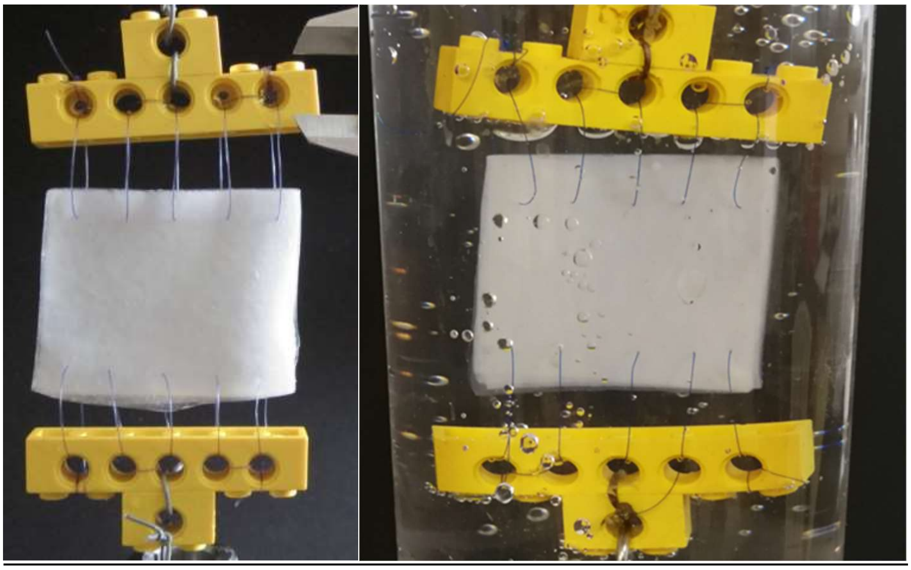

Supplementary Figure S10. Suturability of electropsun PVA membranes.

<u>**Staristical Analysis**</u>

Supplementary Table S1: Explanation of the significance levels calculated using unpaired t-test anaylsis.

Interpretation of significance levels		
significant	$p < 0.0001$	
	$p < 0.0005$	
	$p < 0.001$	
	$p < 0.005$	
	$p < 0.05$	
not significant	$p > 0.05$	

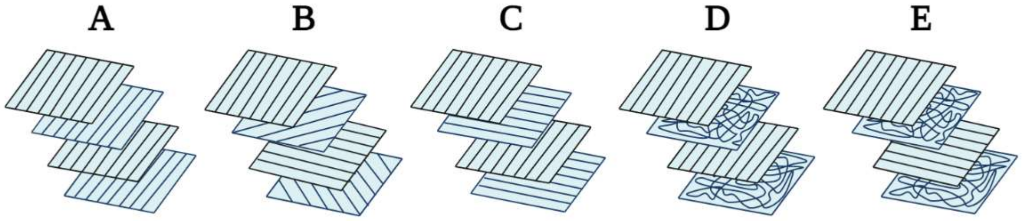

Supplementary Table S2: Analysis of the multilayered vertical samples.

Multilayered vertical samples					
	A	B	C	D	E
A					
B					
C					
D					
E					

Supplementary Table S3: Analysis of the multilayered horizontal samples.

**Multilayered horizontal samples**					
	A	B	C	D	E
A					
B					
C					
D					
E					

Supplementary Table S4: Analysis of the multilayered, sutured vertical samples.

**Multilayered, sutured vertical samples**					
	A	B	C	D	E
A					
B					
C					
D					
E					

Supplementary Table S5: Analysis of the multilayered, sutured horizontal samples.

**Multilayered, sutured horizontal samples**					
	A	B	C	D	E
A					
B					
C					
D					
E					
